# Clinical Resistome Screening of 1,110 *Escherichia coli* Isolates Efficiently Recovers Diagnostically Relevant Antibiotic Resistance Biomarkers and Potential Novel Resistance Mechanisms

**DOI:** 10.3389/fmicb.2019.01671

**Published:** 2019-08-13

**Authors:** Carsten Volz, Jonas Ramoni, Stephan Beisken, Valentina Galata, Andreas Keller, Achim Plum, Andreas E. Posch, Rolf Müller

**Affiliations:** ^1^Helmholtz International Laboratory, Department of Microbial Natural Products (MINS), Helmholtz Institute for Pharmaceutical Research Saarland (HIPS), Helmholtz Centre for Infection Research (HZI), Saarbrücken, Germany; ^2^Ares Genetics GmbH, Vienna, Austria; ^3^Chair for Clinical Bioinformatics, Saarbrücken, Germany; ^4^German Center for Infection Research (DZIF), Braunschweig, Germany

**Keywords:** functional metagenomics, antibiotic resistance, high-throughput screening, biomarkers, bioinformatics, biostatistics, next-generation sequencing

## Abstract

Multidrug-resistant pathogens represent one of the biggest global healthcare challenges. Molecular diagnostics can guide effective antibiotics therapy but relies on validated, predictive biomarkers. Here we present a novel, universally applicable workflow for rapid identification of antimicrobial resistance (AMR) biomarkers from clinical *Escherichia coli* isolates and quantitatively evaluate the potential to recover causal biomarkers for observed resistance phenotypes. For this, a metagenomic plasmid library from 1,110 clinical *E. coli* isolates was created and used for high-throughput screening to identify biomarker candidates against Tobramycin (TOB), Ciprofloxacin (CIP), and Trimethoprim-Sulfamethoxazole (TMP-SMX). Identified candidates were further validated *in vitro* and also evaluated *in silico* for their diagnostic performance based on matched genotype-phenotype data. AMR biomarkers recovered by the metagenomics screening approach mechanistically explained 77% of observed resistance phenotypes for Tobramycin, 76% for Trimethoprim-Sulfamethoxazole, and 20% Ciprofloxacin. Sensitivity for Ciprofloxacin resistance detection could be improved to 97% by complementing results with AMR biomarkers that are undiscoverable due to intrinsic limitations of the workflow. Additionally, when combined in a multiplex diagnostic *in silico* panel, the identified AMR biomarkers reached promising positive and negative predictive values of up to 97 and 99%, respectively. Finally, we demonstrate that the developed workflow can be used to identify potential novel resistance mechanisms.

## Introduction

Microbial infections and antimicrobial resistance (AMR) have become a major healthcare challenge causing projected to claim up to 10 million casualties annually by 2050 (O’Neill, 2016). The rapidly spreading AMR problem is largely fueled by the indiscriminate use of antibiotics which is mostly due to the lack of diagnostic information on the causative pathogens and associated resistance patterns at the time of treatment. The situation is further exacerbated by pharmaceutical companies withdrawing funds from development of novel anti-infectives (Butler et al., 2013; Simpkin et al., 2017) leading to a lack of novel structural classes (May, 2014; O’Neill, 2016), particularly of those active against high priority gram-negative pathogens (WHO, 2017; Kingwell, 2018). With an increasing likelihood of multidrug-resistant infections, diagnostic information about antimicrobial resistance prior to treatment becomes more and more important. Current molecular diagnostics solutions using polymerase chain reaction (PCR) can deliver this information based on a few AMR biomarkers within less than 1–2 h; however, they largely fail at providing comprehensive AMR profiles. Next-generation sequencing (NGS) in contrast can provide genetic antibiotic resistance profiles (Gordon et al., 2014; Walker et al., 2015; Fabre et al., 2018; Ruesen et al., 2018) and is increasingly applied for clinical microbiology and infection control applications (Deurenberg et al., 2017). Moreover, the use of NGS for surveillance studies was also recently recommended by the European Commission for Disease Control and prevention (ECDC, 2016) resulting in a continuous increase in countries routinely using NGS as part of national infection surveillance programs (ECDC, 2018). Regardless of the detection technology used, accurate molecular diagnosis of AMR depends on validated AMR biomarkers. However, conventional approaches to biomarker validation require deep genetic and phenotypic profiling work as well as laborious functional validation work, hence limiting the application of biomarkers for emerging resistance mechanisms into clinical practice.

Functional metagenomics is a multi-step experimental approach that uses expression libraries encoding isolated DNA of mixed microbial populations for the transformation of a relevant host microorganism. Transformed clones can subsequently be selected for a broad range of phenotypes of interest, e.g., enzymatic activities such as lipo-, haemo- or hydrolytic activities (Henne et al., 2000; Rondon et al., 2000; Ottoni et al., 2017). This potentially results in the discovery of novel candidate enzymes that are encoded in the original microbial population. This shotgun metagenomics approach is also applicable to screen for AMR determinants using various microbial consortia as starting material (Allen et al., 2009; Lang et al., 2010; Moore et al., 2011; Torres-Cortes et al., 2011; Van Der Helm et al., 2017; Marathe et al., 2018) and was also employed for determining the taxonomic or phylogenetic origin of microbial consortia in environmental samples by recovering phylogenetic marker genes (Yung et al., 2009). In this study, we investigate whether a functional metagenomics approach can efficiently recover diagnostically relevant antibiotic resistance biomarkers as well as identify potential novel resistance mechanisms from actual clinical isolates.

Here, we describe the development, implementation, and validation of a functional metagenomics screening workflow to rapidly identify and validate AMR biomarkers from clinical isolates for the antibiotics Tobramycin (TOB), Ciprofloxacin (CIP), and Trimethoprim-Sulfamethoxazole (TMP-SMX). This study builds on GEAR-base, a comprehensive collection of isolated DNA, whole-genome sequences and phenotypic antibiotic resistance data of 11,087 clinical isolates for 18 main pathogenic bacterial species which were collected in Australia, Japan, North America, and Europe across three decades (Galata et al., 2019). Combining whole-genome information with quantitative AMR phenotypes, GEAR-base allows for detailed evaluation of AMR biomarker candidates using diagnostic performance indicators such as sensitivity, specificity, and positive and negative predictive values. While this work primarily describes the development and universal applicability of a functional metagenomics screening workflow to effectively recover and validate known AMR biomarkers from clinical samples, we additionally demonstrate that the developed method is equally applicable for identification of potential novel resistance mechanisms.

## Materials and Methods

### Strains, Plasmids, and Primers

Wildtype strains *E. cloni* 10G and *E. coli* DH10B were used for functional metagenomics screening, and all strains, plasmids, and primers used in this study are described in detail in Supplementary Tables S1–3.

### Determination of Minimal Inhibitory Concentration for Wildtype Strains

Wildtype strains *E. cloni* 10G and *E. coli* DH10B were inoculated in 20 ml of LB medium and incubated o/n at 37°C. The cell density was then adjusted to OD600 = 1 using fresh LB medium. Subsequently, aliquots of 100 μl of the liquid culture were plated out on LB agar plates containing increasing amounts of the antibiotics TOB (Sigma), CIP (Fluka), and TMP-SMX (Sigma). After 24 h of incubation at 37°C, the agar plates were examined for grown colonies.

MIC in liquid LB medium was determined as follows. Aliquots of 5 ml of LB medium containing increasing amounts of antibiotic were inoculated with an overnight culture of the test strains to an initial OD600 of 0.01. After incubation o/n at 37°C, the OD600 was measured.

### Generation of Metagenomic Library and Quality Control

The metagenomic library was prepared by Apharmas (American Pharma Source LLC, Rockville, MD 20850, USA). For this, the genomic DNA of 1,110 clinical *E. coli* isolates from GEAR-base was pooled and digested with *Sau*3A (New England Biolabs, Massachusetts, USA). The digested DNA was size-fragmented twice by agarose gel electrophoresis, and the DNA fragments were selected, purified, and ligated into the pACYC184 vector, pre-cut with *Bam*HI and Shrimp Alkaline Phosphatase (New England Biolabs, Massachusetts, USA). Ligated plasmids were transformed into *E. coli* DH5-alpha (New England Biolabs, Massachusetts, USA) and *E. cloni* 10G supreme (Lucigen, Wisconsin, USA). About 50% of the cells of the primary library were used to extract plasmid DNA, which was subsequently column purified forming the metagenomic plasmid library. For QC, PCR was conducted by using pACYC184 specific primers (see Supplementary Table S2). Additionally, an aliquot of the plasmid library was subjected to NGS to further assess and verify homogenous coverage of the pooled 1,110 *E. coli* genomes.

### Direct Cultivation and Screening of the Primary Metagenomic Library

Thorough screening for AMR biomarkers was performed based on the primary library and the plasmid library after retransformation (compare section “Screening Using the Metagenomic Plasmid Library”). Direct screening for resistant clones in the primary metagenomic library was performed. Therefore, aliquots of 100–500 μl (1.5 × 10^4^ cfu per ml) of cells of the pooled primary library were plated on agar plates. LB agar was used for a screening on TOB and CIP (Miller, 1972). Chemically defined agar was used for screening on TMP-SMX as LB agar might contain dihydropteroate, dihydrofolate, or tetrahydrofolate, thereby circumventing the inhibition of dihydropteroate-synthase (DHPS) or dihydrofolate-reductase (DHFR) by TMP-SMX. Growth media were supplemented with the desired antibiotics in concentrations of 2x and 4x MIC, respectively. M9 medium or agar was further supplemented with Valine, Isoleucine, and Leucin each at 100 μg/ml final concentration. As a negative control, an equal amount of *E. cloni* 10G cells harboring an empty vector pACYC184 was treated in the same way. After an incubation o/n at 37°C, plates were inspected for growing resistant colonies. Resistant colonies were used to inoculate 2 ml LB medium supplemented with the same concentration of antibiotics used in the screening on agar plates and grown o/n at 37°C. Subsequently, grown cultures were used for plasmid extraction. Obtained plasmids were finally retransformed into *E. coli* DH10B *via* electroporation according to standard procedure (Dower et al., 1988). For this, strain DH10B (instead of *E. cloni* 10G) was used to ensure different genomic background in order to exclude intrinsic resistance. Selection was again performed on plates containing the same concentration of antibiotics. Subsequently, plasmids of surviving colonies were extracted, pooled, and sequenced by NGS in order to obtain the DNA sequence of the cloned inserts.

### Screening Using the Metagenomic Plasmid Library

In order to identify AMR determinants using plasmid DNA of the primary library, commercially available chemically competent cells of *E. cloni* 10G (Lucigen, Wisconsin, USA) were used. Cells were transformed with plasmid DNA by heat shock treatment as described in the manufacturer’s protocol. Cells which obtained a plasmid conferring resistance to the antibiotic of interest were again selected on agar plates supplemented with the desired antibiotics in concentrations of 2x and 4x MIC, respectively. Selection on agar plates, plasmid isolation, retransformation of plasmids, and sequencing were performed like described for the direct screening of the primary metagenomic library.

### Antimicrobial Resistance Biomarker Validation on Single-Gene Level

AMR biomarker candidates encoded on extracted resistance plasmids, which were identified *in silico* from contigs obtained by NGS, were cloned as *Bam*HI-*Kpn*I (5′ to 3′ orientation) fragments into the pUC19 plasmid digested with *Bam*HI and *Kpn*I. Alternatively, prioritized genes were cloned as *Bam*HI-blunt fragments (5′–3′) into pUC19 digested with *Bam*HI and *Sma*I. Selection of recombinant plasmids was performed on 150 μg/ml ampicillin in *E. coli* DH10B according to the resistance cassette *bla* on the plasmid backbone of pUC19. The sequence of the cloned inserts of the resulting plasmids was verified by Sanger sequencing using primers annealing upstream and downstream of the multiple cloning site of pUC19. These derivatives of pUC19 enabled expression of the candidate genes under the control of a constitutive, modified version of the T7A1 promoter (the sequence of the promoter fragment is provided in Supplementary Figure S2; plasmids and cloned inserts are listed in Supplementary Table S3; Deuschle et al., 1986). In case of the validation of identified resistance markers for TMP-SMX, co-expression of two resistance determinants was necessary two enable resistance development of transformants against both antibiotics TMP-SMX on M9 agar. Therefore, the gene of the identified sulfonamide-resistant DHPS Sul1 was cloned as a *Bam*HI-fragment into pACYC184 pre-cut with *Bam*HI. The recombinant plasmid was subsequently co-transformed together with the respective pUC19 derivatives containing single genes of various identified DHFR enzymes which had been cloned into pUC19 like described above. Transformants were selected on 150 μg/ml ampicillin (for selection of pUC19 derivatives) and 34 μg/ml chloramphenicol (for selection of pACYC184 derivative).

Sequenced plasmids were retransformed in *E. cloni* 10G *via* electroporation, and the transformants were analyzed for emerging resistance on different concentrations of the respective antibiotics (2- to 10-fold MIC). As a negative control, *E. cloni* 10G cells transformed with empty pUC19 or both, pUC19 and pACYC184 were analyzed for their susceptibility in presence of equal concentrations of the antibiotics.

### DNA Sequencing

#### Sanger Sequencing

DNA sequencing of sub-cloned single genes was performed using Sanger sequencing using primers as indicated in Supplementary Table S2.

#### Next-Generation Sequencing

Cloned inserts in putative AMR conferring plasmids of the metagenomic library, as identified by screening under antibiotic selection pressure, were sequenced by NGS. Therefore, equal amounts of all extracted plasmids from survivor colonies were pooled. Similarly, sequencing of genomic DNA from strains *E. cloni* 10G and *E. coli* DH10B was performed by NGS. Genomic DNA was isolated by standard laboratory procedures (Maniatis et al., 1982). Quality and purity of the genomic DNA was verified by agarose gel electrophoresis, as well as spectrometric measurement at 280, 260, and 230 nm using a NanoDrop 2000C spectrophotometer (ThermoFisher Scientific, Massachusetts, USA). DNA library preparation was performed at Microsynth AG using Nextera XT Library (Illumina, USA) according to the manufacturer’s protocol. The resulting DNA was pooled, and NGS was performed on MiSeq 2×150, v2, (Illumina, USA).

### Sequence Data Analysis

#### Metagenomic Plasmid Library Quality Control

Uniform genome coverage of the plasmid library was verified through NGS. An aliquot of the metagenomic plasmid library was sequenced on MiSeq, v2, 2 × 150 bp using Illumina Nextera XT library preparation yielding 1 Gb output. FASTQ reads were quality trimmed using Trimmomatic v0.38 (Bolger et al., 2014) in paired end-mode with default settings. Read quality was assessed with FastQC (Andrews, 2010) v0.11.8 prior to sequence alignment against the *E. coli* pan-genome with bowtie2 v2.3.4.3 (Langmead and Salzberg, 2012). The pan-genome 1,110 clinical *E. coli* isolates as generated and described by Galata et al. (2019), resulting in a final pan-genome consisting of 939 contig genome assemblies, was used for quality control of the plasmid library and statistical analysis of biomarker candidates from the plasmid pools. Gene coverage of the metagenomic plasmid library alignment against the pan-genome was calculated *via* samtools v1.9 (Li et al., 2009).

#### Functional Metagenomic Screening

Sequenced raw reads were processed as described above. After alignment to the *E. coli* pan-genome with bowtie2 v2.3.4.3 (alignment parameters set to “end-to-end” and “sensitive”), plasmid genes were recalled from the alignments *via* average gene depth, compiled, and extracted with samtools v1.9 (Li et al., 2009). Per-nucleotide read depths were averaged per gene and thresholded by an average depth of 30x to rule out potential contaminant genomic DNA. The resulting candidate gene lists comprising all ORFs exhibiting a minimum length of 60 amino acids (aa) were functionally annotated using both, a blast analysis BLASTP (Altschul et al., 1990) and a protein domain analysis InterProScan 5 (Jones et al., 2014). Subsequently, candidate genes were evaluated for diagnostic performance based on matched genotype-phenotype data in GEAR-base.

To assess the theoretical diagnostic performance of the complete set of known biomarkers putatively encoded in the pooled plasmid library, the entire *E. coli* pan-genome was queried with ResFinder (commit 510e159) (Zankari et al., 2012). Such identified biomarkers by ResFinder were subsequently used to estimate the diagnostic performance of ResFinder for each of the three compounds.

#### Analysis of Wildtype Reference Strains

Sequence reads from wildtype strains *E. cloni* 10G and *E. coli* DH10B and DH5-alpha were quality checked [FastQC v0.11.8 (Andrews, 2010)] and trimmed [Trimmomatic v0.38 (Bolger et al., 2014)] before the *de novo* assembly with SPAdes v3.12.0 (Bankevich et al., 2012) and feature annotation with prokka v1.13.3. (Seemann, 2014). Near completeness to public reference genomes for DH5-alpha (ID: NZ_CP025520.1) and DH10B (ID: NC_010473.1) was checked with Quast v.5.0.2 (Gurevich et al., 2013). No reference genome was used for *E. cloni* 10G. Absence of recovered plasmid genes was checked against contig assemblies through nucleotide BLAST (blastn v2.7.1+).

#### Diagnostic Performance Evaluation

Diagnostic performance metrics for each candidate biomarker selected from the functional metagenomic screening were calculated based on the pan-genome gene presence-absence matrix and matched phenotypic resistance profiles for the 1,110 isolates. Performance summary metrics derived from the confusion matrix were calculated under the assumption that gene presence confers resistance. *In silico* candidate marker panel statistics were derived under the assumption that the presence of any single marker of a marker panel confers resistance.

Marker panel statistics based on biomarkers detected with ResFinder were treated equivalently: individual genes from the pan-genome that were identified as resistance conferring against the respective antibiotic according to the ResFinder database were aggregated in a panel under the assumption that the presence of a single gene confers the resistance phenotype.

Subsequent candidate marker selection for experimental validation was based on a minimum true positive threshold of 10 isolates per candidate marker and their positive predictive values (PPVs). Candidate markers with high PPVs and functional annotations that indicate a mechanistic link between a marker and the observed resistance phenotype was further validated *in vitro* on single gene level.

#### *De novo* Assembly of Cloned Library gDNA of Plasmid Pools

In order to obtain information about the number and continuous sequence of different cloned library DNA fragments in the pools of pACYC184 derivatives isolated on TOB, TMP-SMX, and CIP, paired reads from NGS sequencing were used in a *de novo* assembly of such contigs using the Geneious Prime version 2019.0.4 software package and the Geneious assembler^1^.

## Results

### Functional Metagenomics Screening Using Clinical Isolates

**Figure 1:**
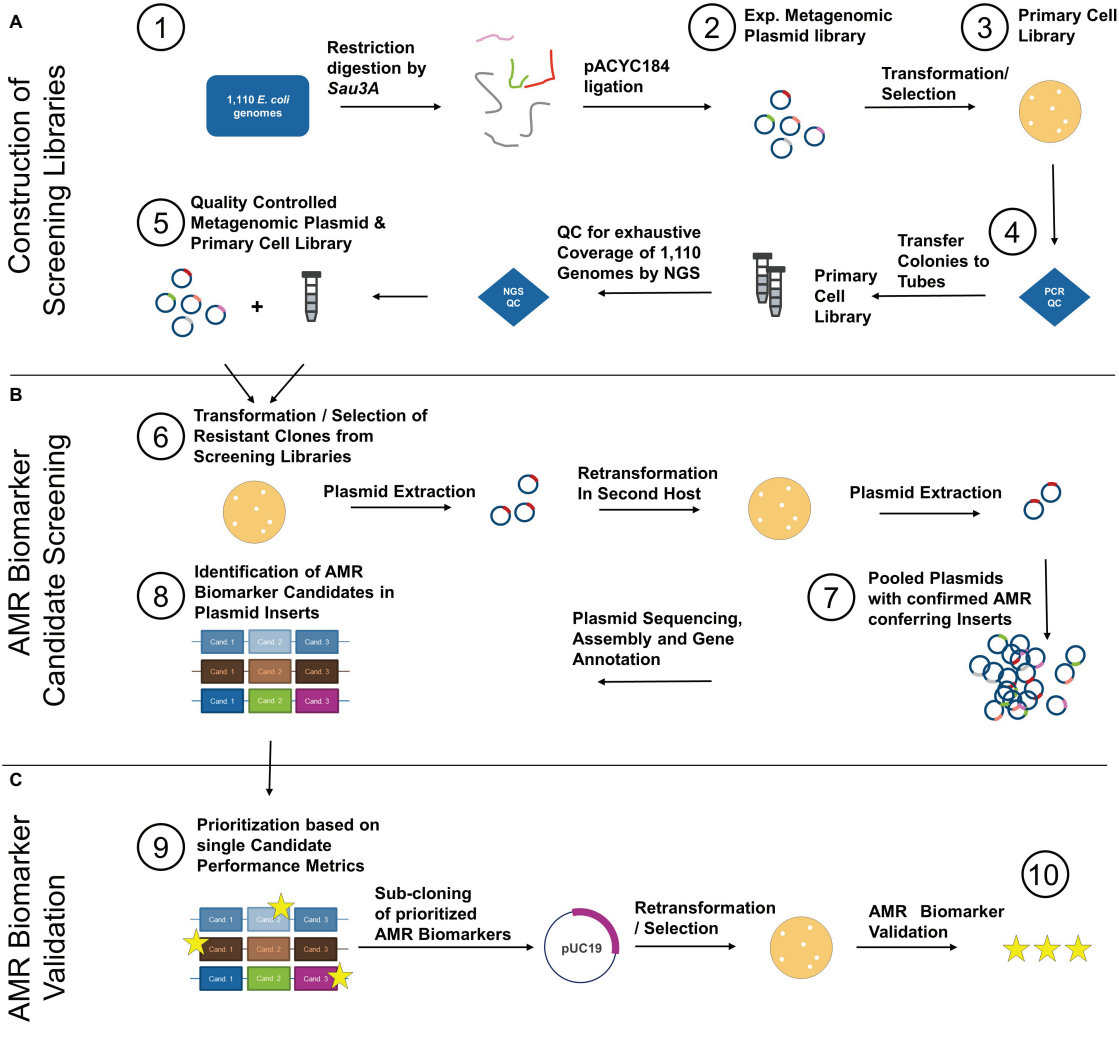
Isolated DNA from 1,110 clinical *E. coli* of the GEAR-base biobank was pooled and restriction digested by Sau3A (1). Resulting fragments were cloned into pACYC184 yielding an experimental metagenomic plasmid library (2). After transformation/selection on chloramphenicol (3), the primary cell library was quality controlled for recombination ratio by colony PCR (see Supplementary Figure S1) (4). Half of the primary cell library was subjected to plasmid extraction and the plasmid library was quality controlled by NGS to ensure homogenous coverage of the 1,110 *E. coli* genomes (5). After QC, the plasmid library was used to transform competent *E. coli*, which was selected on TOB, TMP-SMX, and CIP (6). Additionally, cells of the primary cell library were directly cultivated and selected on the same antibiotics. Survivor colonies were plasmid extracted and plasmids retransformed into *E. coli* of different genetic background. Subsequently, resistant clones were plasmid extracted and the plasmids of all clones were pooled per antibiotic (7) and sequenced by NGS. NGS data were analyzed, and all identified AMR biomarker candidates (8) were prioritized based on diagnostic performance metrics calculated from matched genotype-phenotype information from the 1,110 *E. coli* (9). Prioritized AMR biomarker candidates were sub-cloned on pUC19 vector and expression plasmids harboring prioritized candidates were transformed into *E. coli* to causally confirm mediation of resistance. Functionally validated AMR biomarkers were subsequently combined per antibiotic and the performance metrics of such *in silico* diagnostic multiplex panel was determined (10).

The developed functional metagenomics screening workflow comprising of (A) construction of the screening library, (B) AMR biomarker screening, and (C) AMR biomarker validation is illustrated in Figure 1.

### Metagenomic Library Quality Control

A metagenomic plasmid library from 1,110 clinical *E. coli* isolates was constructed resulting in a primary cell library of 42 vials containing 1 ml cell suspension with a titer of 1.5 × 10^5^ cfu/ml. The quality of the primary cell library was determined *via* PCR for recombination efficiency (see Supplementary Figure S1) revealing a > 83% recombination ratio with 93% of colonies bearing an insert size of 2–5 kb in the respective pACYC184 derivative. The insert size was determined by balancing mean prokaryotic gene lengths of around 1 kb and experimental economic considerations. An insert size range from 2 to 5 kb theoretically enabled the efficient capture of the vast majority of prokaryotic genes. Half of the volume of the primary cell library was plasmid extracted forming a transformation grade plasmid library with a concentration of 183 μg/ml in a total volume of 0.7 ml.

**Figure 2:**
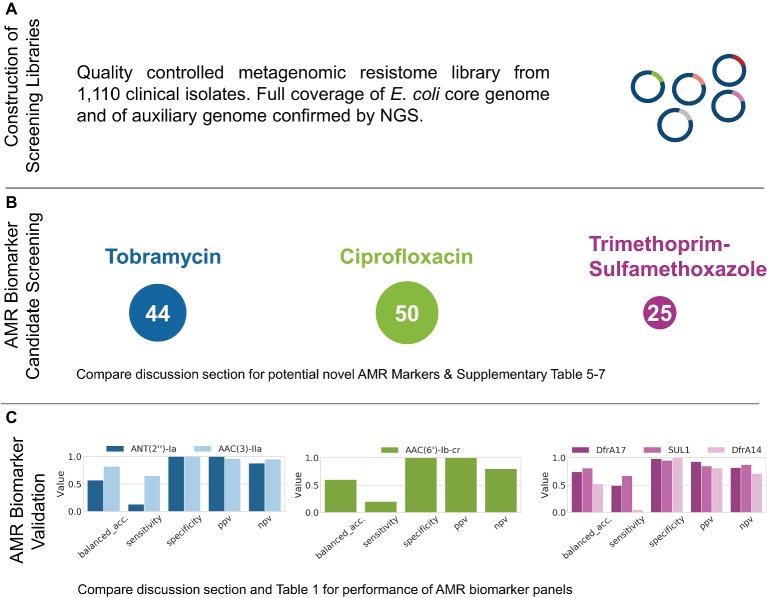
Results obtained from the functional metagenomics screening workflow described in Figure 1. After Step 5, the representative coverage of the 1,110 *E. coli* genomes by the metagenomic library was confirmed by NGS **(A)**. AMR screening after Step 8 identified 44, 50, and 25 AMR marker candidates for TOB, CIP, and TMP-SMX, respectively **(B)**. After further prioritization based on matched genotype-phenotype data for the isolates used for library generation, the proposed workflow was applied to successfully recover and validate AMR markers for all antibiotics tested (Step 10, **C**).

To confirm homogenous coverage of 1,110 *E. coli* genomes in the plasmid library, the plasmid library was sequenced by NGS and mapped against the pan-genome constructed from the contig assemblies of the 1,110 clinical isolates. The entire pan-genome was largely recovered from the sequenced aliquot, and a total of 4,464 genes were found at high sequencing depth > 30x, substantially exceeding the core genome size of the 1,110 *E. coli* isolates amounting to 3,390 genes (Figure 2A).

### Functional Metagenomic Screening

Prior to using wildtype isolates *E. cloni 10G* and *E. coli DH10B* for functional metagenomics and selection on the antibiotic of interest, their corresponding minimum inhibitory concentrations (MICs) were determined (see Supplementary Table S4). Subsequently, aliquots of cells of the primary metagenomic library were directly selected on agar plates supplemented with TOB, CIP, or TMP-SMX. In addition, the metagenomic plasmid library was transformed in *E. cloni 10G* and selected on agar plates supplemented with TOB, CIP, and TMP-SMX. Plasmids of survivor colonies were extracted and retransformed into *E. coli DH10B* to confirm resistance development. Afterwards, extracted plasmids of survivors were pooled per antibiotic and sequenced by NGS. This resulted in a pool of 44, 50, and 25 identified candidates with an average sequencing depth of >30x for TOB, CIP, and TMP-SMX, respectively (Figure 2B).

### Diagnostic Performance of Identified Antimicrobial Resistance Markers and *in silico* Panels

AMR biomarker candidates identified from survivor colonies after Step 8 were subsequently selected for further evaluation based on performance metrics obtained from matched genotype-phenotype data, ranked for highest PPV and true positive counts exceeding a minimum threshold of 10 (Figure 2B, see Supplementary Tables S5–7). The genes *ant (2″)-Ia* and *aac(3)-IIa* were selected as best TOB candidates and were cloned into pUC19 for single gene validation. When transformed into *E. coli* 10G, this led to >10x increase in MIC towards TOB. As for TMP-SMX, prioritization leads to co-expression of *sul1* together with *dfrA17* and *dfrA14*, respectively. This again led to high-level resistance (>10x MIC) in both cases. As for CIP candidates ranking, we selected and cloned *aac(6′)-lb-cr* into pUC19 which resulted in resistance of transformants toward CIP > 5 MIC (all Figure 2C, compare Supplementary Tables S5–7).

Subsequently, the validated AMR markers were combined in *in silico* diagnostic panels, and their performance metrics were calculated. Such designed AMR panels could mechanistically explain 77% of resistance phenotypes for TOB, 76% for TMP-SMX, and 20% for CIP (Figure 1C, Table 1). When complementing CIP results with known point mutations in *gyrA*, which are undetectable with the applied method (further elaborated in the discussion), the overall sensitivity could be increased further. Interestingly, the transporter protein *EmrE* was identified and highly ranked for predicitive performance for all antibiotics tested (see Seq ID C3830-194_04604 in Supplementary Tables S5–7). Upon inclusion of *EmrE*, the negative predictive value of the *in silico* panels reached ≥90% across all three antibiotics (99% for TOB, 90% for CIP and TMP-SMX, see Table 1).

**Table 1:** Performance metrics of *in silico* diagnostic panels.

*In silico* AMR biomarker panel	Accuracy	Balanced accuracy	Sensitivity	Specificity	PPV	NPV	TP	FP	TN	FN
TOB panel	0.97	0.88	0.77	1.00	0.97	0.97	95	3	813	28
TOB panel + EmrE	0.84	0.89	0.94	0.83	0.45	0.99	116	140	676	7
TOB ResFinder	0.97	0.90	0.80	0.99	0.97	0.97	99	3	813	24
CIP panel	0.84	0.60	0.20	1.00	0.97	0.83	38	1	747	153
CIP panel + EmrE	0.81	0.74	0.63	0.85	0.52	0.90	121	110	638	70
CIP panel + gyrA83/87	0.95	0.96	0.97	0.95	0.82	0.99	185	41	707	6
CIP ResFinder	0.84	0.60	0.20	1.00	0.98	0.83	39	1	747	152
TMP-SMX panel	0.88	0.85	0.76	0.94	0.83	0.90	211	42	619	67
TMP-SMX panel + EmrE	0.89	0.85	0.77	0.94	0.84	0.90	213	42	619	65
TMP-SMX Resfinder	0.84	0.88	0.98	0.78	0.65	0.99	273	145	516	5

*PPV, positive predictive value; NPV, negative predictive value; TP, true positive; FP, false positive; TN, true negative; FN, false negative*.

The predictive performance of AMR marker panels was further compared to the theoretical maximum performance based on known AMR biomarkers – as identified by ResFinder – present in the pan-genome that are putatively encoded in the plasmid pool. Those pan-genome panels achieved greater sensitivity for TOB (80%) and TMP-SMX (98%) than the corresponding validated AMR marker panels. For CIP, sensitivity is identical between ResFinder identified and validated AMR marker panels, given that known point mutations in *gyrA* are equally undetectable with ResFinder as with the applied method. While the negative predictive value exceeds 90% for TMP-SMX, its ResFinder identified marker panel captures more false positives than the validated AMR marker panels, resulting in a lower positive predictive value of 65%. No significant predictive difference can be seen between the Resfinder identified and validated TOB panels.

### Detailed Analysis of Resistance Conferring Plasmid Insert Contigs

Complementing the high-throughput AMR biomarker screening and largely automated evaluation workflow with detailed manual analyses, all plasmid insert contigs (selected for high coverage and pACYC184 flanks) were additionally *de novo* assembled and analyzed by functional annotation. In case of TOB, this revealed four unique contigs. Functional annotation identified four different aminoglycoside-modifying enzymes, one encoded on each contig: Aminoglycoside N(3)-acetyltransferase [*aac(3)-IId*], aminoglycoside 3′′-phosphotransferase type I (*aph3′-1*), aminoglycoside N-acetyltransferase [*aac(3)-IIa*] and aminoglycoside nucleotidyltransferase [*ant (2″)-Ia*]. As for TMP-SMX, this resulted in three unique contigs, each of the contigs containing a DHPS (*sul1* or *sul2*) and a DHFR gene (*dfrA17*, *folA*, or *dfrA14*). Hence, we could successfully identify the AMR conferring mechanisms of all plasmids extracted from survivor colonies for TOB and TMP-SMX. As for CIP however, 12 unique contigs were assembled but only two known resistance determinants were identified [CIP-modifying aminoglycoside-acetyltransferase Aac(6′)-Ib-cr and the quinolone resistance protein QnrB19]. In a first attempt to identify the respective genes conferring resistance to CIP, single genes of four additional contigs were expressed in *E. cloni* 10G (two aminoglycoside-modifying adenylyltransferases AadA and AadB, a putative hydrolase named Hydro, as well as a tunicamycin-resistance protein Tun). However, none of these could be confirmed to confer resistance to CIP. To better explain potential novel CIP AMR mechanisms based on predicted functions of encoded putative proteins and their possible relation to the resistance phenotype, an in-depth functional analysis of all ORFs with a minimal size of 60 amino acids (aa) was performed. This led to the identification of a total number of 174 open reading frames (ORFs) with most of them being hypothetical proteins with no similarity to known protein sequences (70.7%) followed by transposases/integrases (11.4%), conserved hypothetical proteins (8.6%), and diverse modifying enzymes (6.3%) (Table 2). The detailed analysis of these potential novel AMR mechanisms is currently subject of further experimental studies.

**Table 2:** Functional prediction of putative proteins on ciprofloxacin contigs encoding unknown resistance determinants.

Predicted function	No. of ORFs	Rel. abundance (%)
Transport functions	4	2.3
Modifying enzymes	11	6.3
Regulatory functions	1	0.6
Transposases*	14	8.0
Integrases*	6	3.4
Conserved hypothetical proteins	15	8.6
Hypothetical proteins	123	70.7

* *Also partial*.

## Discussion

Historically, the discovery of AMR and AMR biomarkers was largely based on tedious forward genetics experiments to identify causative genes and genetic mutations. Here, we describe a universally applicable, functional metagenomics workflow and demonstrate its potential to effectively recover diagnostically relevant biomarkers as well as identify potential novel resistance mechanisms for Tobramycin (TOB), Trimethoprim-Sulfamethoxazole (TMP-SMX), and Ciprofloxacin (CIP). The three antibiotics were selected to cover (1) different modes of action, (2) antibiotics with known gene and point-mutation mediated resistance, and (3) antibiotic combination drugs to ensure broad applicability of the developed workflow. Additionally, selection was limited to antibiotics included in GEAR-base with at least 100 resistant clinical *E. coli* isolates to allow for statistical evaluation of performance characteristics.

### Tobramycin

Applying the developed workflow, we successfully identified AMR markers for aminoglycoside resistance covering three classes of aminoglycoside-modifying enzymes. Besides those, only a few additional mechanisms of aminoglycoside resistance are known: First, increased efflux, second reduced uptake ability, and third, altered ribosomal binding sites, the latter two being caused by genetic mutations. The screening workflow successfully identified the *EmrE* transporter for increasing the likelihood of resistance; the remaining two mechanisms are arguable of minor clinical relevance. While reduced uptake of aminoglycosides only leads to a moderate increase in resistance, different aminoglycosides bind to different sites on the ribosome. Hence, high-level resistance towards various aminoglycosides is only achieved if different sites on all encoded copies of the respective ribosomal genes would be mutated simultaneously because prokaryotes encode multiple copies of many ribosomal subunits or proteins. Exceptions are *Mycobacterium* spp. and *Borrelia* spp. For a comprehensive overview of Tobramycin resistance mechanisms, see Krause et al. and references therein (Krause et al., 2016). Since low to medium level resistance mechanisms were not observed in our screening, this might either mean that these mutations causing this type of resistance are not present in the metagenomic library or that the conferred resistance level is too low to be observed. The latter point is substantiated by the *in silico* ResFinder identified panel for Tobramycin, which – based on known resistance-conferring markers – could only increase detected resistance phenotypes by 3% when compared to the regular TOB panel – excluding non-specific EmrE-mediated efflux. The main resistance mechanisms were successfully identified by the workflow.

### Trimethoprim-Sulfamethoxazole

Resistance to TMP-SMX is mediated by multiple mechanisms related to the two target enzymes of Trimethoprim and Sulfamethoxazole, DHFR and DHPS, respectively (Miovic and Pizer, 1971; Sköld, 2001; Jonsson et al., 2003). These mechanisms include (1) DHFR exhibiting a natural intrinsic insensitivity toward Trimethoprim, (2) spontaneous susceptibility-lowering mutations in intrinsically susceptible DHFR, (3) increased expression of DHFR, and (4) horizontal acquisition of resistant DHFR genes (Huovinen et al., 1995; Pikis et al., 1998; Brochet et al., 2008). Sulfonamide resistance is primarily mediated by the presence of sulfonamide-resistant DHPS enzymes *Sul1*, *Sul2*, and *Sul3*. Intrinsic resistance toward Trimethoprim and sulfonamides has also been reported as a result of intrinsic low permeability of the cell membrane in several species; however, these mechanisms seem not to be horizontally transmissible like resistant variants of DHFR and DHPS. In our approach, we could successfully identify three known trimethoprim-resistant variants of DHFR (DfrA17, FolA, and DfrA14) and two out of three known sulfonamide-resistant DHPS enzymes (Sul1 and Sul2). A sequence analysis of the variants of DHFR and DHPS in this study revealed no single nucleotide polymorphism variants, and we assume that resistance was due to higher abundance of the respective proteins causing a titration effect. Analogous to our findings in Tobramycin, addition of low- to medium-level resistance mechanisms may increase sensitivity at the cost of specificity. In contrast to Tobramycin resistance, TMP-SMX resistance appears to be mediated by a larger number of mechanisms, potentially explaining the larger gap in sensitivity between the validated AMR marker panels and the theoretical ResFinder identified panels.

### Ciprofloxacin

Resistance toward fluoroquinolones such as CIP is mainly based on chromosomal mutations that alter DNA gyrase and topoisomerase IV, alteration of porins types, and amounts thereof (Jacoby, 2005). Additionally, plasmid-mediated resistance determinants like aminoglycoside acetyltransferase AAC(6′)-Ib-cr and the pentapeptide family proteins Qnr were reported (Strahilevitz et al., 2009). As for this study, we only recovered plasmid-borne resistance mechanisms in single gene expression experiments, whereas other prominent resistance factors for CIP such as altered DNA gyrase or topoisomerase IV remained uncovered. However, this finding can be explained by the mode of action of CIP: Both, DNA gyrases and topoisomerases are DNA remodeling enzymes and as such targets of CIP. Binding of Ciprofloxacin to those targets causes stabilization of the covalent enzyme-DNA cleavage-complex (in a process called “poisoning”). This results in DNA breaks and initiates a cascade of events that ultimately lead to cell death (Drlica et al., 2009; Kohanski et al., 2010). Hence, we reason that a titration effect as likely observed for TMP-SMX resistance is not possible for CIP as remaining native enzymes lead to lethal phenotypes also in the additional presence of plasmid-encoded enzymes harboring resistance conferring mutations. Therefore, the herein used methodology might bear an intrinsic limitation in discovering resistance elements that cannot be recovered due to native proteins causing lethal phenotypes that cannot be rescued by expressing resistant determinants. However, we believe to have recovered the main plasmid-mediated resistance determinants as corroborated by the almost identical performance between the validated AMR marker and theoretical ResFinder identified panels.

Interestingly, a considerable amount of the assembled contigs obtained from NGS data of pooled pACYC184 derivatives did not encode known resistance determinants toward CIP. Concerned of potential CIP artifact resistances, all resistance plasmids were retransformed in other genetic backgrounds, which again lead to reproducible resistance phenotypes. This led us to conclude that several yet unknown resistance determinants or combinations thereof must be encoded on those fragments. Analyzed for predicted functions, most of the identified open reading frames encode for hypothetical proteins with a yet unknown function in AMR. To unravel their role in AMR, further characterization experiments are required.

## Conclusion

Here, we describe a functional metagenomics screening workflow for rapid and cost-effective discovery and functional validation of AMR biomarkers to facilitate translation of novel AMR biomarkers from clinical samples to diagnostic applications. Results of this study are based on a metagenomics screening library of unprecedented scale, combining 1,110 globally collected clinical *E. coli* isolates. Recovering the most prominent, diagnostically relevant AMR biomarkers for three antibiotics of distinct mode of action, Tobramycin, Trimethoprim-Sulfamethoxazole, and Ciprofloxacin, underscores the universal applicability of the workflow. As highlighted by Morel et al. (2016), the use of AMR biomarkers has the potential not only to accelerate and lower the cost of antibiotics drug development but also to guide effective treatment options as part of a companion diagnostics (Richards et al., 2015). The benefit of molecular diagnostics in enabling successful treatment outcomes is further underlined by a large meta-study showing that the use of diagnostic biomarkers in clinical trials is associated with a nearly 3-fold higher success rate for novel anti-infectives and infectious disease treatment options (Wong et al., 2019). Based on those findings, we conclude that the continuous and effective discovery, validation, and diagnostic use of AMR biomarkers will remain of utmost importance to enable early informed therapy and effectively fighting AMR. Functional metagenomics combined with NGS-based screening has already been shown to be a powerful tool for the identification of AMR associated biomarkers in environmental samples. However, to the best of our knowledge, it has so far not been shown that AMR markers can also efficiently be recovered from actual clinical samples to subsequently inform relevant diagnostic applications. Hence, using 1,110 *E. coli* isolates with matched whole-genome sequences and antimicrobial resistance profiles, we demonstrate for the first time, that (1) a metagenomics screening approach can effectively recover diagnostic AMR markers from clinical samples and (2) those AMR markers, when subsequently combined in a panel achieve very promising diagnostic performance parameters. We therefore conclude that the methodology described in this study will be an invaluable tool to facilitate rapid validation of novel AMR biomarkers for diagnostic use.

## Data Availability

Raw sequence reads have been submitted to the Sequence Read Archive (SRA) as part of BioProject PRJNA521456. The BioProject includes the sequenced plasmid library, mutant plasmid pools selected with Tobramycin, Trimethoprim-Sulfamethoxazole, and Ciprofloxacin and sequenced laboratory strains of DH5-alpha, DH10B, and *E. cloni* 10G. Genome assemblies of the sequenced laboratory strains have been deposited with GenBank: GCA_006352225.1 (DH5-alpha), GCA_006352235.1 (DH10B), and GCA_006363855.1 (*E. cloni* 10G).

## Author Contributions

AEP, RM, JR, CV, and AP designed the study. CV performed experiments. SB, JR, and CV analyzed data. AEP, JR, CV, and SB wrote the manuscript. All authors reviewed and approved the manuscript.

### Conflict of Interest Statement

AEP, SB, JR, and AP were employed by the company Ares Genetics.

The remaining authors declare that the research was conducted in the absence of any commercial or financial relationships that could be construed as a potential conflict of interest.

## Footnotes

^1^https://www.geneious.com

## Abbreviations

AMR: Antimicrobial resistance
CIP: Ciprofloxacin
NGS: Next-generation sequencing
TOB: Tobramycin
TMP-SMX: Trimethoprim-Sulfamethoxazole.
