# Supplementary material for: Clinical Resistome Screening of 1,110 Escherichia coli Isolates Efficiently Recovers Diagnostically Relevant Antibiotic Resistance Biomarkers and Potential Novel Resistance Mechanisms
Source: Front Microbiol. 2019 Aug 13;10:1671. doi: 10.3389/fmicb.2019.01671 (PMC6700387; doi:10.3389/fmicb.2019.01671)
Supplement: Supplementary file 1 [file Data_Sheet_1.PDF]

Supplementary Material

1 Supplementary Data

1.1 Supplementary Figures

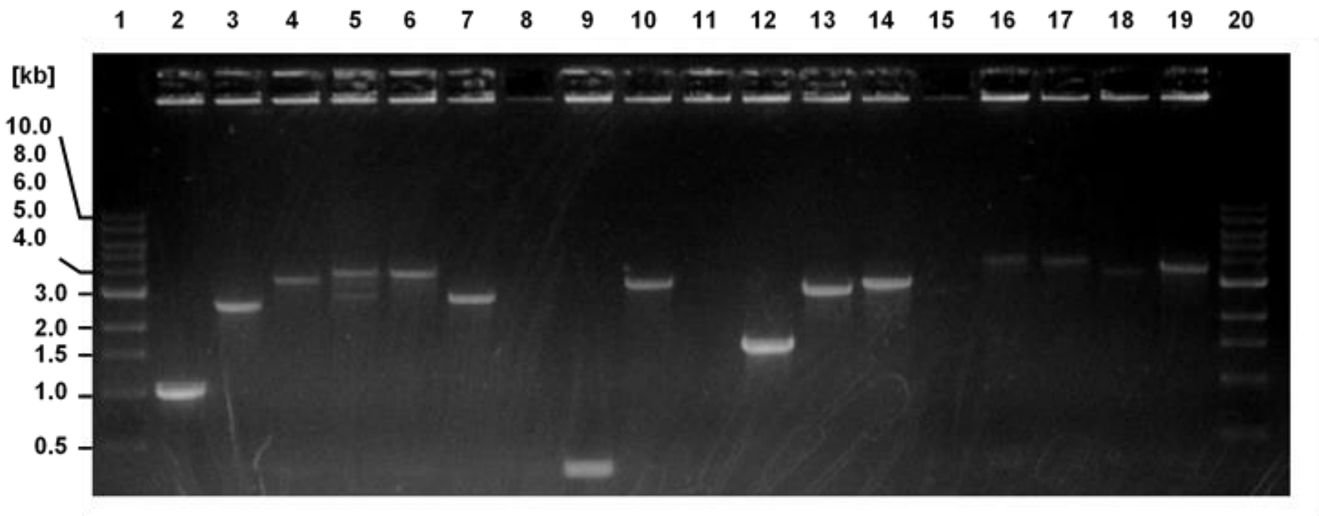

Supplementary Figure 1. Plasmid library quality control was conducted by PCR using pACYC184 specific primers. 18 colonies were randomly picked from a titration plate and targeted for colony PCR. As shown on the picture of an 1 % agarose gel, 15 of 18 colonies have inserts (recombination ratio >83%), which insert sizes of the 14 tested colonies (93%) are between 2 to 5 kB (most >3kb). 2 colonies (Lane 7 and Lane 10) were not PCR-amplified, possibly due to GC-rich or other technical reasons. Only Lane 9 indicated a vector only. Lane 1 & Lane 20: NEB 1kb molecular weight marker. Lane 2 – 19_ colonies #1 to 18 (note: Lane 14 is ~3 kb but faint due to low resolution).

5'-ATCAAAAAGAGTATTGACTTAAAGTCTAACCTATAGGATACTTACAGCCATAGGAGG*TGATTAT*-3'

Supplementary Figure 2. Sequence of the modified version of promoter T7A1 in 5' to 3' orientation. The original core region of the T7A1 promoter is highlighted in black. The modified region at the 3'-end is highlighted in red and comprises a modified ribosomal binding site (RBS; underlined) as well as a 7 bp spacer region (distance of RBS to downstream cloned start codon of respective ORF (italics).

1.2 Supplementary Tables

Supplementary Table 1. Strains used in this study

Strain	Origin
E. cloni 10G	Lucigen, Wisconsin, USA
E. coli DH10B	New England Biolabs, Massachusetts, USA

Supplementary Table 2. Primers used in this study

**Name**	**Sequence (5' – 3')**	**Used for**	**Reference**
pACF	TTC TCG GAG CAC TGT CCG AC	QC of library	This study
pACR	AAG GAA TGG TGC ATG CAA G	QC of library	This study
toco3F	**GAGGGATCC***ATCAAAAAGAGTATT GACTTAAAGTCTAACCTATAGGATA CTTACAGCCATAGGAGGTGATTAT*A TGCATACGCGGAAGGCAATAACG	cloning of candidate genes in pUC19	This study
toco3R	**CCGGTACC**CTAACCGGAAGGCTC GCAAGAGC	cloning of candidate genes in pUC19	This study
toco4F	**GAGGGATCC***ATCAAAAAGAGTATT GACTTAAAGTCTAACCTATAGGATA CTTACAGCCATAGGAGGTGATTAT*A TGGACACAACGCAGGTCACATTG ATAC	cloning of candidate genes in pUC19	This study
toco4R	**CCGGTACC**TTAGGCCGCATATCG CGACCTG	cloning of candidate genes in pUC19	This study
coco1F	**GAGGGATCC***ATCAAAAAGAGTATT GACTTAAAGTCTAACCTATAGGATA CTTACAGCCATAGGAGGTGATTAT*T TGAAAATATCATTGATTTCTGCAG TG	cloning of candidate genes in pUC19	This study
coco1R	**CCGGTACC**TTAGCCTTTTTCCA AATCTGGTATG	cloning of candidate genes in pUC19	This study
sul1F	**GAGGGATCC***ATCAAAAAGAGTATT GACTTAAAGTCTAACCTATAGGATA CTTACAGCCATAGGAGGTGATTAT*A TGGTGACGGTGTTCGGCATTC	cloning of candidate gene in pACYC184	This study
sul1R	**GAGGGATCC**CTAGGCATGATCTA ACCCTCGG	cloning of candidate gene in pACYC184	This study
coco3F	**GAGGGATCC***ATCAAAAAGAGTATT GACTTAAAGTCTAACCTATAGGATA CTTACAGCCATAGGAGGTGATTAT*A TGAGAACCTTGAAAGTATCATTG ATAGC	cloning of candidate genes in pUC19	This study
coco3R	**CCGGTACC**TTAACCCTTTTTCCA AATTTGATAGCAATAG	cloning of candidate genes in pUC19	This study
cico1aacF	**GAGGGATCC***ATCAAAAAGAGTATT GACTTAAAGTCTAACCTATAGGATA CTTACAGCCATAGGAGGTGATTAT*A	cloning of candidate genes in pUC19	This study

	TGAGCAACGCAAAAACAAAGTTAG		
cico1aacR	TTAGGCATCACTGCGTGTTCG	cloning of candidate genes in pUC19	This study
cico1aadAF	**GAGGGATCC***ATCAAAAAGAGTATTGACTTAAAGTCTAACCTATAGGATACTTACAGCCATAGGAGGTGATTAT*ATGGGTGAATTTTTCCCTGCACAAG	cloning of candidate genes in pUC19	This study
cico1aadAR	**CCGGTACC**TCAACGCAAGATTCTCTCAATCGTTGC	cloning of candidate genes in pUC19	This study
cico2aadBF	**GAGGGATCC***ATCAAAAAGAGTATTGACTTAAAGTCTAACCTATAGGATACTTACAGCCATAGGAGGTGATTAT*ATGGACACAACGCAGGTCACATTGATAC	cloning of candidate genes in pUC19	This study
cico2aadBR	**CCGGTACC**TTAGGCCGCATATCGCGACCTG	cloning of candidate genes in pUC19	This study
cico5-tunF	**GAGGGATCC***ATCAAAAAGAGTATTGACTTAAAGTC*<br>**GAGGGATCC***ATCAAAAAGAGTATTGACTTAAAGTCTAACCTATAGGATACTTACAGCCATAGGAGGTGATTAT*ATGATAATCTGGATCAACGGACCTTTCG	cloning of candidate genes in pUC19	This study
cico5-tunR	**CCGGTACC**TCAGGTGCGCTGATCGAGTCTG	cloning of candidate genes in pUC19	This study
cico5-5-76F	**GAGGGATCC***ATCAAAAAGAGTATTGACTTAAAGTCTAACCTATAGGATACTTACAGCCATAGGAGGTGATTAT*TTGCAGTTTAGAGGAGATATCGCGATG	cloning of candidate genes in pUC19	This study
cico5-5-76R	**CCGGTACC**TCATACGTCACCCACCGTTTGTTGG	cloning of candidate genes in pUC19	This study
cico7hydroF	**GAGGGATCC***ATCAAAAAGAGTATTGACTTAAAGTCTAACCTATAGGATACTTACAGCCATAGGAGGTGATTAT*TTGCTAGCGACCTGCGTCAGC	cloning of candidate genes in pUC19	This study
cico7hydroR	**CCGGTACC**TGAGCCCGAAGTGGCGAGC	cloning of candidate genes in pUC19	This study
pUCF	GCCAGTGAATTCGAGCTCGG	sequencing of pUC19 inserts	LGC Genomics
pUCR	TGCCTGCAGGTCGACTCTAG	sequencing of pUC19 inserts	LGC Genomics

Supplementary Table 3. Plasmids used in this study

Name	Cloned gene	GenBank no. of closest homologue	Similarity to homologue (aa)	Origin (homologue)	Expected resistance	Origin of cloned gene (NGS contig)	Origin of plasmid backbone	Reference (plasmid)
pUC19-toco3	*aac(3)-IIa*	AYD37473.1	100%	*K. pneumoniae*	TOB	TOB contig 3	pUC19	This study
pUC19-toco4	*ant (2‘ ‘)-Ia*	AJY59716.1	100%	*S. enterica*	TOB	TOB contig 4	pUC19	This study
pUC19-coco1dfr	*dfrA17*	AXZ05400.1	100%	*E. coli* AR_0086	TMP-SMX	TMP-SMX contig 1	pUC19	This study
pUC19-coco3dfr	*dfrA14*	ASO50230.1	100%	*S. enterica*	TMP-SMX	TMP-SMX contig 3	pUC19	This study
pACYC-sul1	*sul1*	AXZ05301.1	100%	*E. coli* AR_0086	TMP-SMX	TMP-SMX contig 1	pACYC	This study
pUC19-cico1aac	*aac(6')-Ib-cr*	ACD56150.1	100%	*E. coli*	CIP	CIP contig 1	pUC19	This study
pUC19-cico1aadA	*aadA*	BBF51267.1	100%	*E. coli*	CIP	CIP contig 1	pUC19	This study
pUC19-cico2aadB	*aadB*	AMG48254.1	100%	*Achromobacter xylosoxidans*	CIP	CIP contig 2	pUC19	This study
pUC19-cico7hydro	n.a.	n.a.	n.a.	n.a.	CIP	CIP contig 7	pUC19	This study
pUC19-cico5tun	n.a.	n.a.	n.a.	n.a.	n.a.	n.a.	pUC19	This study
pUC19	n.a.	n.a.	n.a.	n.a.	n.a.	n.a.	pUC19	(1)
pACYC 184	n.a.	n.a.	n.a.	n.a.	n.a.	n.a.	pACYC 184	(2)

Supplementary Table 4. MIC values determined in this study

Antibiotic	Abbreviation	MIC (µg/ml)
Tobramycin	TOB	3
Ciprofloxacin	CIP	0,007
Trimetoprim-Sulfamethoxazol	TMP-SMX	5

Supplementary table 5. Diagnostic performance metrics of top candidates ranked by PPV identified by sequencing TOB plasmid pools. Chosen candidates are highlighted in green. Performance characteristics calculated based on 939 evaluated contig assemblies. (TP = True Positives, FP = False Positives, TN = True Negatives, FN = False Negatives)

seq_id	compound	accuracy	balanced_accuracy	sensitivity	specificity	ppv	npv	tp	fp	tn	fn	depth
C3830-194_04779	TOB	0,91	0,64	0,28	1,00	1,00	0,90	34	0	816	89	75
C5125-5604_04775	TOB	0,89	0,57	0,13	1,00	1,00	0,88	16	0	816	107	3423
C5127-5764_03951	TOB	0,87	0,51	0,02	1,00	1,00	0,87	2	0	816	121	92
C5127-5767_05124	TOB	0,87	0,50	0,01	1,00	1,00	0,87	1	0	816	122	478
C3830-194_04780	TOB	0,95	0,82	0,65	1,00	0,96	0,95	80	3	813	43	4247
C3830-281_04962	TOB	0,89	0,59	0,18	1,00	0,96	0,89	22	1	815	101	1601
C3830-281_04990	TOB	0,91	0,65	0,30	1,00	0,95	0,90	37	2	814	86	151
C3830-194_04707	TOB	0,94	0,77	0,54	1,00	0,94	0,94	67	4	812	56	2892
C5125-5604_04773	TOB	0,88	0,54	0,08	1,00	0,91	0,88	10	1	815	113	876
C5129-5927_04941	TOB	0,87	0,51	0,02	1,00	0,67	0,87	2	1	815	121	2530
C5125-5586_04758	TOB	0,87	0,51	0,02	1,00	0,60	0,87	3	2	814	120	205
C3830-193_04669	TOB	0,87	0,54	0,09	0,99	0,48	0,88	11	12	804	112	759
C3830-193_04668	TOB	0,86	0,53	0,08	0,98	0,42	0,88	10	14	802	113	669
C3830-193_04670	TOB	0,86	0,53	0,07	0,98	0,39	0,88	9	14	802	114	448
C3830-194_04604	TOB	0,81	0,74	0,65	0,83	0,36	0,94	80	140	676	43	2260
C3830-193_04062	TOB	0,79	0,78	0,76	0,80	0,36	0,96	93	163	653	30	3225
C3831-289_03906	TOB	0,85	0,55	0,13	0,96	0,35	0,88	16	30	786	107	716
C3830-193_04399	TOB	0,77	0,77	0,76	0,78	0,34	0,96	94	183	633	29	103
C3830-193_04398	TOB	0,77	0,77	0,76	0,77	0,34	0,96	94	186	630	29	33
C3830-194_04804	TOB	0,81	0,65	0,42	0,87	0,33	0,91	52	107	709	71	2032
C3831-282_04647	TOB	0,80	0,59	0,30	0,88	0,27	0,89	37	98	718	86	44
C3832-433_04936	TOB	0,82	0,54	0,16	0,92	0,24	0,88	20	62	754	103	6170
C3830-194_01587	TOB	0,61	0,73	0,89	0,57	0,24	0,97	109	353	463	14	39
C3830-194_01588	TOB	0,61	0,73	0,89	0,57	0,24	0,97	109	353	463	14	35
C3830-194_01835	TOB	0,60	0,71	0,85	0,56	0,23	0,96	105	355	461	18	67
C3830-193_04729	TOB	0,60	0,67	0,76	0,57	0,21	0,94	94	349	467	29	42

C3833-506_04749	TOB	0,81	0,53	0,15	0,91	0,20	0,88	18	73	743	105	33
C3830-194_04017	TOB	0,51	0,59	0,69	0,49	0,17	0,91	85	418	398	38	47
C3830-193_04772	TOB	0,46	0,55	0,68	0,42	0,15	0,90	84	471	345	39	4878
C5126-5645_04670	TOB	0,87	0,50	0,00	1,00	0,00	0,87	0	1	815	123	365
C5129-5980_04530	TOB	0,87	0,50	0,00	1,00	0,00	0,87	0	1	815	123	2463
C5130-6102_04826	TOB	0,87	0,50	0,00	1,00	0,00	0,87	0	1	815	123	45
C5131-6128_04679	TOB	0,87	0,50	0,00	1,00	0,00	0,87	0	1	815	123	594
C5131-6180_04446	TOB	0,87	0,50	0,00	1,00	0,00	0,87	0	1	815	123	120
C5132-6258_04488	TOB	0,87	0,50	0,00	1,00	0,00	0,87	0	1	815	123	168
C5132-6275_04710	TOB	0,87	0,50	0,00	1,00	0,00	0,87	0	1	815	123	170
C5134-6482_04565	TOB	0,87	0,50	0,00	1,00	0,00	0,87	0	1	815	123	1745
C5126-5711_04634	TOB	0,87	0,50	0,00	1,00	0,00	0,87	0	2	814	123	210
C5129-5947_04841	TOB	0,87	0,50	0,00	1,00	0,00	0,87	0	2	814	123	680
C5129-5947_04842	TOB	0,87	0,50	0,00	1,00	0,00	0,87	0	2	814	123	62
C5129-5966_04777	TOB	0,87	0,50	0,00	1,00	0,00	0,87	0	2	814	123	54
C3832-398_04497	TOB	0,87	0,50	0,00	1,00	0,00	0,87	0	3	813	123	536
C3892-113_04926	TOB	0,86	0,50	0,00	0,99	0,00	0,87	0	7	809	123	105
C5125-5549_04663	TOB	0,86	0,49	0,00	0,99	0,00	0,87	0	11	805	123	100

Supplementary table 6. Diagnostic performance metrics of top candidates ranked by PPV identified by sequencing TMP-SMX plasmid pools. Chosen candidates are highlighted in green. Performance characteristics calculated based on 939 evaluated contig assemblies. (TP = True Positives, FP = False Positives, TN = True Negatives, FN = False Negatives)

seq_id	compound	accuracy	balanced_accuracy	sensitivity	specificity	ppv	npv	tp	fp	tn	fn	depth
C3892-153_03589	TMP-SMX	0,71	0,51	0,02	1,00	1,00	0,71	5	0	661	273	217
C5127-5764_03951	TMP-SMX	0,71	0,50	0,01	1,00	1,00	0,71	2	0	661	276	48
C5134-6427_04810	TMP-SMX	0,71	0,50	0,00	1,00	1,00	0,70	1	0	661	277	35
C3830-194_04603	TMP-SMX	0,82	0,71	0,42	0,99	0,94	0,80	118	7	654	160	1526
C3830-194_04602	TMP-SMX	0,84	0,74	0,49	0,98	0,93	0,82	135	10	651	143	836
C3830-194_04604	TMP-SMX	0,87	0,81	0,67	0,95	0,85	0,87	187	33	628	91	1170
C3830-194_04606	TMP-SMX	0,86	0,80	0,65	0,95	0,85	0,87	182	33	628	96	179

C3830-194_04605	TMP-SMX	0,87	0,81	0,67	0,95	0,85	0,87	187	34	627	91	1251
C3830-193_04062	TMP-SMX	0,88	0,85	0,77	0,93	0,83	0,90	213	43	618	65	382
C3831-374_04868	TMP-SMX	0,71	0,52	0,05	1,00	0,81	0,71	13	3	658	265	784
C3832-433_04936	TMP-SMX	0,75	0,60	0,22	0,97	0,76	0,75	62	20	641	216	6279
C3830-194_04670	TMP-SMX	0,82	0,76	0,62	0,90	0,73	0,85	172	65	596	106	470
C5129-5927_04941	TMP-SMX	0,71	0,50	0,01	1,00	0,67	0,71	2	1	660	276	3435
C3830-194_04671	TMP-SMX	0,79	0,75	0,64	0,86	0,66	0,85	178	93	568	100	738
C5129-5947_04841	TMP-SMX	0,70	0,50	0,00	1,00	0,50	0,70	1	1	660	277	665
C5129-5947_04842	TMP-SMX	0,70	0,50	0,00	1,00	0,50	0,70	1	1	660	277	47
C3832-379_04689	TMP-SMX	0,70	0,51	0,04	0,98	0,40	0,71	10	15	646	268	118
C3830-194_04017	TMP-SMX	0,59	0,63	0,71	0,54	0,39	0,82	198	305	356	80	41
C3830-191_02841	TMP-SMX	0,30	0,50	1,00	0,00	0,30	0,00	278	661	0	0	4975
C3830-191_02842	TMP-SMX	0,30	0,50	1,00	0,00	0,30	0,00	278	661	0	0	5801
C3830-191_02843	TMP-SMX	0,30	0,50	1,00	0,00	0,30	0,00	278	661	0	0	5965
C3830-191_02846	TMP-SMX	0,30	0,50	1,00	0,00	0,30	0,00	278	661	0	0	5273
C3830-191_02847	TMP-SMX	0,30	0,50	1,00	0,00	0,30	0,00	278	661	0	0	1143
C5129-5980_04530	TMP-SMX	0,70	0,50	0,00	1,00	0,00	0,70	0	1	660	278	2894
C5134-6482_04565	TMP-SMX	0,70	0,50	0,00	1,00	0,00	0,70	0	1	660	278	2066

Supplementary table 7. Diagnostic performance metrics of top candidates ranked by PPV identified by sequencing CIP plasmid pools. Chosen candidates are highlighted in green. Performance characteristics calculated based on 939 evaluated contig assemblies. (TP = True Positives, FP = False Positives, TN = True Negatives, FN = False Negatives)

seq_id	compound	accuracy	balanced_accuracy	sensitivity	specificity	ppv	npv	tp	fp	tn	fn	depth
C5129-5927_04941	CIP	0,80	0,51	0,02	1,00	1,00	0,80	3	0	748	188	5782
C5127-5764_03951	CIP	0,80	0,51	0,01	1,00	1,00	0,80	2	0	748	189	660
C3830-281_04990	CIP	0,84	0,60	0,20	1,00	0,97	0,83	38	1	747	153	3748
C3830-281_04962	CIP	0,82	0,55	0,11	1,00	0,91	0,81	21	2	746	170	1110
C3830-194_04780	CIP	0,86	0,68	0,38	0,99	0,87	0,86	72	11	737	119	6253

C3830-194_04707	CIP	0,85	0,65	0,32	0,99	0,86	0,85	61	10	738	130	2283
C3830-281_04991	CIP	0,82	0,57	0,16	0,99	0,83	0,82	30	6	742	161	700
C5125-5604_04775	CIP	0,81	0,53	0,06	0,99	0,75	0,81	12	4	744	179	6884
C3830-194_04602	CIP	0,87	0,75	0,56	0,95	0,74	0,89	107	38	710	84	117
C3830-194_04603	CIP	0,86	0,71	0,47	0,95	0,72	0,88	90	35	713	101	218
C3892-111_04262	CIP	0,80	0,51	0,02	1,00	0,60	0,80	3	2	746	188	279
C3830-193_04399	CIP	0,83	0,82	0,80	0,83	0,55	0,94	153	124	624	38	1940
C3830-193_04062	CIP	0,81	0,76	0,69	0,83	0,52	0,91	132	124	624	59	7561
C3830-194_04604	CIP	0,80	0,71	0,58	0,85	0,50	0,89	110	110	638	81	6570
C3830-194_04605	CIP	0,80	0,71	0,58	0,85	0,50	0,89	110	111	637	81	183
C3830-194_01804	CIP	0,76	0,71	0,61	0,80	0,44	0,89	117	148	600	74	100
C3830-194_04804	CIP	0,78	0,62	0,37	0,88	0,44	0,84	70	89	659	121	6313
C3830-194_01805	CIP	0,76	0,70	0,60	0,80	0,43	0,89	114	151	597	77	113
C3830-194_01835	CIP	0,68	0,76	0,91	0,62	0,38	0,96	173	287	461	18	1063
C3832-398_04497	CIP	0,80	0,50	0,01	1,00	0,33	0,80	1	2	746	190	3872
C3831-310_04844	CIP	0,77	0,52	0,10	0,94	0,31	0,80	20	44	704	171	49
C3830-202_03772	CIP	0,67	0,61	0,52	0,70	0,31	0,85	100	221	527	91	197
C3832-433_04936	CIP	0,76	0,53	0,13	0,92	0,30	0,81	25	57	691	166	7205
C3830-194_04017	CIP	0,59	0,67	0,80	0,53	0,30	0,91	153	350	398	38	282
C3833-506_04749	CIP	0,76	0,52	0,14	0,91	0,29	0,81	26	65	683	165	118
C3831-315_04806	CIP	0,79	0,50	0,02	0,99	0,27	0,80	4	11	737	187	315
C3831-294_04854	CIP	0,71	0,53	0,24	0,83	0,26	0,81	46	129	619	145	31
C3831-282_04647	CIP	0,73	0,52	0,18	0,87	0,26	0,81	35	100	648	156	43
C3830-194_04495	CIP	0,46	0,61	0,86	0,36	0,26	0,91	165	480	268	26	352
C3830-193_04759	CIP	0,44	0,59	0,83	0,34	0,24	0,89	159	492	256	32	218
C3830-193_04772	CIP	0,49	0,57	0,71	0,44	0,24	0,85	135	420	328	56	6631
C3831-289_04646	CIP	0,70	0,50	0,18	0,83	0,21	0,80	34	126	622	157	32
C3830-191_02841	CIP	0,20	0,50	1,00	0,00	0,20	0,00	191	748	0	0	5011
C3830-191_02842	CIP	0,20	0,50	1,00	0,00	0,20	0,00	191	748	0	0	6034
C3830-191_02843	CIP	0,20	0,50	1,00	0,00	0,20	0,00	191	748	0	0	6408
C3830-191_02846	CIP	0,20	0,50	1,00	0,00	0,20	0,00	191	748	0	0	5912
C3830-191_02847	CIP	0,20	0,50	1,00	0,00	0,20	0,00	191	748	0	0	1248
C3830-193_04699	CIP	0,55	0,46	0,30	0,62	0,17	0,78	58	286	462	133	38
C3831-292_04867	CIP	0,78	0,50	0,01	0,98	0,13	0,80	2	13	735	189	662

C3831-298_04793	CIP	0,80	0,50	0,00	1,00	0,00	0,80	0	1	747	191	3656
C3831-298_04794	CIP	0,80	0,50	0,00	1,00	0,00	0,80	0	1	747	191	4280
C5129-5980_04530	CIP	0,80	0,50	0,00	1,00	0,00	0,80	0	1	747	191	4859
C5131-6180_04446	CIP	0,80	0,50	0,00	1,00	0,00	0,80	0	1	747	191	923
C5132-6258_04488	CIP	0,80	0,50	0,00	1,00	0,00	0,80	0	1	747	191	1352
C5133-6366_04819	CIP	0,80	0,50	0,00	1,00	0,00	0,80	0	1	747	191	206
C5134-6482_04565	CIP	0,80	0,50	0,00	1,00	0,00	0,80	0	1	747	191	6239
C5129-5947_04841	CIP	0,79	0,50	0,00	1,00	0,00	0,80	0	2	746	191	3412
C5129-5947_04842	CIP	0,79	0,50	0,00	1,00	0,00	0,80	0	2	746	191	232
C5126-5654_04866	CIP	0,79	0,50	0,00	1,00	0,00	0,80	0	3	745	191	117
C3833-551_04471	CIP	0,79	0,50	0,00	0,99	0,00	0,80	0	6	742	191	105
